# Transition metal complexes with asymmetric tridentate peripheral organosilyl ligands: their impact in homogeneous catalysis

**DOI:** 10.3389/fchem.2026.1804159

**Published:** 2026-03-23

**Authors:** María Batuecas, Luis A. Oro, Francisco J. Fernández-Álvarez

**Affiliations:** Departamento de Química Inorgánica – Instituto de Síntesis Química y Catálisis Homogénea (ISQCH), Universidad de Zaragoza, Facultad de Ciencias, Zaragoza, Spain

**Keywords:** asymmetric tridentate ligands, homogenous catalysis, organosilyl polydentate ligands, pincer ligands, transition metal-silyl complexes, tridentate silyl ligands

## Abstract

Silyl-based tridentate ligands [SiL_2_]^n−^ (n = 1, 2; L = PR_3_, NR_2_ or other groups) have emerged over the last decades as a versatile class of ligands for transition metal (TM) chemistry. TM–(κ^3^-*Si*,*L*_2_) complexes display unique structural features and reactivity that arise directly from the incorporation of a strongly σ-donating silyl group into the pincer ligand framework. While most studies have focused on systems where silicon occupies the central position of the tridentate scaffold, recent efforts have shifted toward the development of TM complexes in which the silicon atom is located at the periphery of the ligand. This alternative design opens unexplored opportunities to fine-tune electronic and steric properties, offering fresh perspectives for catalyst optimization and the discovery of unprecedented reactivity patterns. This review summarizes and discusses the most recent advances in this emerging area, highlighting strategies for incorporating peripheral silicon into the skeleton of the ligand and exploring its impact on the coordination chemistry, reactivity and catalytic activity of the resulting TM complexes.

## 1 Introduction

In homogeneous catalysis mediated by transition-metal complexes, the ligand plays a decisive role in governing catalytic behavior. Systematic modification of ligand structure, including steric bulk and electronic parameters, allows precise tuning of key steps in the catalytic cycle, such as substrate coordination, activation, and product release. These adjustments can lead to significant improvements in reaction efficiency, selectivity, and catalyst stability. Consequently, the rational design, synthesis, and evaluation of novel ligand frameworks have become central to modern homogeneous catalysis, continually driving advances in catalytic methodologies and practical applications (Lundgren and Stradiotto, 2016).

Over the past few decades, the chemistry of transition-metal complexes featuring multidentate organosilyl ancillary ligands has expanded significantly. These ligands are distinguished by their strong σ-donor properties, as well as by the pronounced *trans*-effect and *trans*-influence exerted by the silyl moiety (Whited and Taylor, 2020). Such features promote the formation of electron-rich metal centers and facilitate access to coordinatively and electronically unsaturated species, which are often crucial intermediates in catalytic cycles. Consequently, organosilyl-based ligand frameworks have emerged as versatile platforms for modulating metal reactivity and enabling new transformations in homogeneous catalysis (Gao et al., 2024; Komuro et al., 2022a).

**FIGURE 1:**
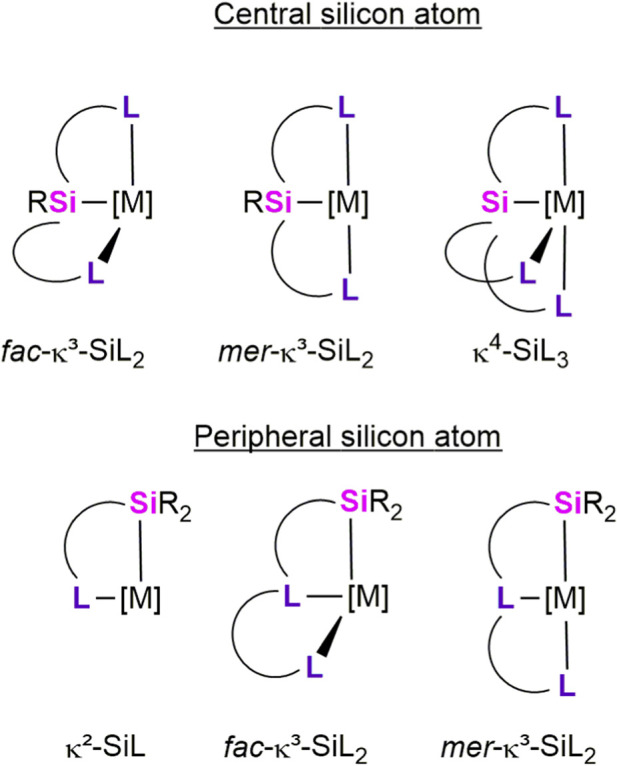
Schematic representation of multidentate silyl ligands.

A wide variety of transition-metal complexes supported by multidentate organosilyl ligands have been reported, encompassing tetradentate κ^4^-Si,L_3_, (Ehrlich et al., 2018; Wagler and Gericke, 2023), tridentate κ^3^-Si,L_2_, (Cabeza and García–Álvarez, 2022; Fernández-Alvarez et al., 2017; Gao et al., 2024; Komuro et al., 2022a; Simon and Breher, 2017; Sola, 2018; Turculet, 2014; Whited, 2021), and bidentate κ^2^-Si,L (Batuecas et al., 2024; Okazaki et al., 2002) coordination modes. In these systems, the silyl fragment (Si) is combined with σ-donor ligands (L) such as *N*-heterocycles, phosphines, *N*-heterocyclic carbenes, thioethers, esters, silyl ethers, and tetrylenes, thereby providing diverse electronic and structural environments at the metal center (Figure 1).

To date, most studies on transition-metal complexes featuring multidentate silyl ancillary ligands have concentrated on tridentate κ^3^-L,Si,L architectures, which have been extensively investigated and summarized in several recent reviews. In contrast, monoanionic bidentate κ^2^-Si,L ligand frameworks have attracted increasing attention in recent years, particularly due to their promising performance in homogeneous hydrosilylation and hydrogenation catalysis (Figure 1). Notably, a number of transition-metal κ^2^-Si,L complexes have demonstrated superior catalytic activity compared to their κ^3^-L,Si,L analogues in transformations such as CO_2_ hydrosilylation and Kumada cross-coupling reactions (Batuecas et al., 2024; Okazaki et al., 2002).

Despite these advances, draws our attention that the chemistry of transition metal complexes with asymmetric tridentate organosilyl ligands remains comparatively underdeveloped, with only a limited number of examples reported to date. In this review, we survey recent advances in the application of transition-metal complexes bearing asymmetric tridentate organosilyl ligands in homogeneous catalysis, highlighting key developments in ligand design, catalytic performance, and mechanistic insight, as well as outlining current challenges and future opportunities in this emerging area.

## 2 Transition-metal complexes with κ^3^-(*Si*,*N*,*N′*) ligands

### 2.1 Borylation catalysts

**FIGURE 2:**
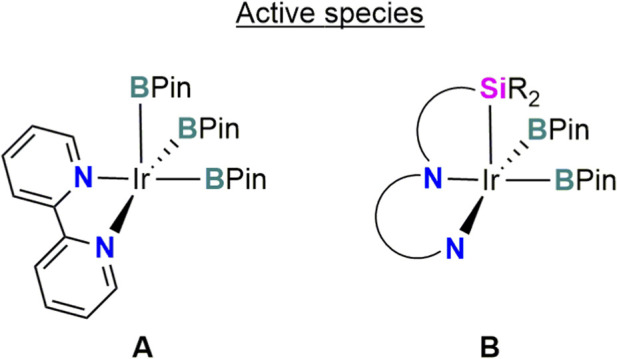
Active species A and B proposed in Ir-catalyzed C–H borylations.

The first transition metal complexes bearing asymmetric (κ^3^-*Si*,*N*,*N′*)-type ligands were reported by Lee et al. (2013). This kind of ligands were carefully designed to favor the C–H activation step in Ir-catalysed borylation reactions. They hypothesized that as for the C–H arene borylation catalyzed by the Ir/bipy system (bipy = 2,2′-bipyridine), the proton transfer from the coordinated arene to the boryl ligand is enhanced by two strong donating boryl ligands of the active species [Ir(Bpin)_3_(κ^2^-bipy)] (A in Figure 2) (Vanchura et al., 2010), a tridentate, dianionic pincer ligand carrying a strong donor comparable to a boryl could perform similarly (Figure 2B).

**SCHEME 1:**
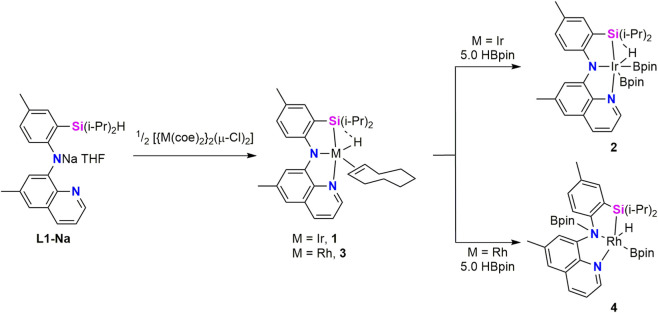
Synthesis of the M-**L1** and M-**L2** (M = Ir, Rh) complexes **1–4**.

In consequence, iridium complexes containing a ligand consisting of a central amido site, a side silyl strong donor and a neutral side donor opposite to the silyl (**L1**) were synthesized and tested in borylation reactions. **Ir-L1** complexes were obtained by reaction of the Na derivative **L1**-Na with half an equivalent of [{Ir(coe)_2_}_2_(μ-Cl)_2_] to yield the Ir(III) hydride complex [Ir(H)(κ^3^-*Si*,*N*,*N′*-**L1**)(coe)] (**1**). Reaction of **1** with HBpin (HBpin = pinacolborane) led to the formation of the related complex with two boryl ligands [Ir(H)(Bpin)_2_(κ^3^-*Si*,*N*,*N′*-**L1)**] (**2**) (Scheme 1). In the ^1^H NMR spectrum of **1** and **2**, the Ir–H hydride resonances appear at δ −21.1 and −14.7 ppm with ^1^ *J*_Si-H_ = 8.0 Hz and 32.0 Hz, respectively, indicating a Si···H interaction, stronger in complex **2** than in **1**. Both complexes were characterized by X-ray diffraction analysis. The Ir–Si bond distances of 2.3573(15) Å (**1**) and 2.4130(14) Å (**2**) fall within the range reported for Ir–silyl bond distances (Batuecas et al., 2024; Padilla et al., 2025a). DFT studies were carried out to determine the location of the Ir-bounded hydrogen atoms. The calculated Si–H bond distances (2.007 Å for **1** and 1.889 Å for **2**) along with experimental Si–H coupling constant values show that complex **1** should be considered as Ir(III) compound with classical silyl and hydride ligands whereas **2** could be viewed either as an Ir(V) silyl/hydride complex or as an Ir(III) derivative with a Si–H ligand.

In 2015, the same research group investigated the chemistry of **L1**-Na with rhodium (Lee et al., 2015). Reaction of **L1**-Na with 0.5 equivalents of [{Rh(coe)_2_}_2_(μ-Cl)_2_] affords the complex [Rh(H)(κ^3^-*Si*,*N*,*N′*-**L1**)(coe)] (**3**) (Scheme 1), which is the rhodium analogue of the previously reported iridium derivative **1**. The ^1^H NMR spectrum of **3** exhibits a hydride resonance at δ −16.9 ppm as a doublet, with a rhodium-hydride coupling constant ^1^ *J*_Rh-H_ = 31 Hz. This resonance is flanked by satellites arising from ^29^Si–H coupling (51 Hz), indicating a stronger Si–H interaction in **3** than that observed for the iridium complex **1**. X-ray diffraction analyses of **3** confirmed the proposed ligand distribution and shows that both complexes, **1** and **3** are isomorphs. Treatment of **3** with 5.0 equiv of HBpin leads to the formation of the Rh(III) complex [Rh(H)(Bpin)(*fac*-κ^3^-*Si*,*N*,*N′*-**L2**)] (**4**) rather than the rhodium species analogous to the Ir complex **2**. Complex **4** is formed by migration of one of the boryl groups onto the central N of the SiNN ligand giving rise to a monoanionic ligand (**L2**) composed by two neutral N donors (quinolone and borylated amine) and the silyl ligand. The ^1^H NMR spectrum of **4** displays the hydride resonance as a doublet at δ −15.0 ppm, with a rhodium–hydride coupling constant of ^**1**^ *J*_Rh-H_ **=** 30 Hz. Notably, no Si–H coupling is observed for complex **4** (Lee et al., 2015). The proposed structure was confirmed by a single-crystal X-ray diffraction study. Experimental data and DFT calculated distances of Rh–H (1.573 Å) and Si–H (2.228 Å) suggest that **4** could be described as a silyl/hydride complex. A detailed study of geometric parameters of M-**L1** and M-**L2** (M = Ir, Rh) was used to describe a continuum between Si–H σ complexes and silyl/hydride derivatives showing the adaptability of Si–H moiety to act either as a neutral 2-electron donor ligand or as two X-type monoanionic ligands depending on the electronic or steric requirements of the metal center. These studies allow to conclude that complex **3** is best described as a Si–H σ-complex, whereas complexes **1** and **4** correspond to a silyl/hydride species; complex **2** lies at the borderline between these limiting descriptions.

Ir(III) complex **1** was proven to be an active chemoselective catalyst for dehydrogenative C–H borylation of terminal alkynes (DHBTA). The catalytic system based on complex **1** (1.0 mol%) in the presence of an excess of HBpin (5.0 eq) exhibits high activity (turnover frequency (TOF) ≈ 600 h^-1^) and excellent selectivity for the borylation of terminal alkynes bearing aryl, alkyl, or silyl substituents, delivering high conversions (96%–99%) and isolated yields (85%–95%) (Lee et al., 2013). Complex **2** shows similar activity than **1** and it was proposed as an intermediate in a preliminary mechanistic proposal similar to that proposed for aromatic C–H borylations. In contrast, the related rhodium complexes **3** and **4** display significantly lower catalytic activity under the same reaction conditions (Lee et al., 2015). This low activity can be related to the facile migration of a boryl ligand to the amide N atom of the ligand on the rhodium complex to form **4** preventing the formation of the hydride/bisboryl species, the potential catalytic intermediate related to the iridium counterpart **2**.

**SCHEME 2:**
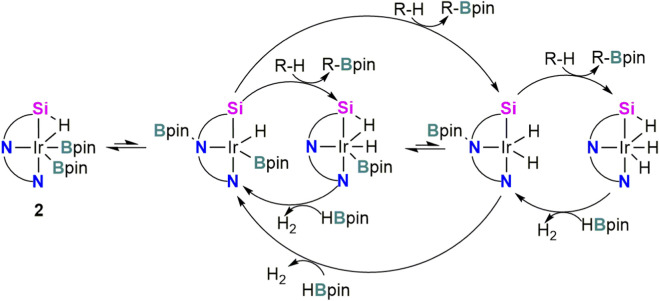
Pathways for the **2**-catalyzed dehydrogenative borylation of terminal alkynes (DHBTA) reported by Ozerov et al.

Some years later, in 2018, this group reported a detailed DFT study of the Ir-**L1** catalyzed DHBTA (Zhou et al., 2018). Three interlinked catalytic cycles with free energy barriers in the range of 16–22 kcal mol^-1^, consistent with the experimentally observed TOF were identified. The fact that cycles have common intermediates and multiple transition states with comparable energies within each catalytic cycle makes difficult to designate a single predominant cycle. However, a common feature of all pathways is the facile migration of the Bpin group between the Ir center and the amido nitrogen of **L1** to generate the corresponding **L2** ligand which electronically and coordinatively activates the Ir center toward C–H oxidative addition (Scheme 2). This migration was experimentally observed in the formation of Rh complex **3** and was calculated slightly endergonic for Ir derivative **1** (2.9 kcal mol^−1^). The Si–H moiety of the ligand was also found a non-innocent feature of the SiNN ligand. The adaptability of the mode of interaction with the metal centre, alternating between oxidative addition and σ-complex enables the Ir center to accommodate structural end electronic changes throughout the progression of different catalytic steps.

With the aim of explaining the high chemoselectivity observed for the **2**-catalyzed DHBTA, Dang *et al.* reported an independent DFT study taking into consideration also competing hydroboration pathways (Chen et al., 2018). The preference for dehydrogenative borylation was attributed to (i) Bpin migration to the N_amido_ of the ligand **L1**; and (ii) stabilization of Ir–H intermediates through interaction with the Si atom of the SiNN pincer ligand, highlighting the key role of ligand cooperativity in controlling chemoselectivity.

**FIGURE 3:**
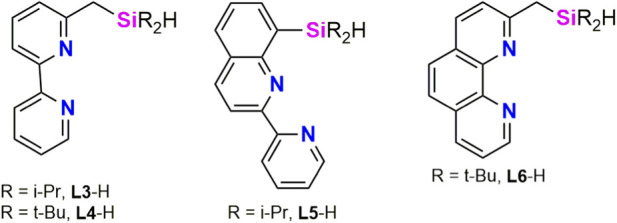
2,2′-bipyridine- and 1,10-phenatroline-based SiNN ligands reported by Kuninobu and collaborators (**L3**-H–**L6**-H).

In 2022, Kuninobu et al. envisioned that LLX-type mer-tridentate ligands with silyl donor groups could be suitable for borylation of unreactive C(sp^3^)–H bonds (Kawazu et al., 2022). κ^3^-Meridional coordination might generate thermally stable iridium intermediates. Furthermore, selectivity could be imposed by bulky substituents on the silyl ligand which could replace one of the boryl ligand of the tris(boryl) species, assumed to be the catalytic species in borylation reactions. Four new bipyridine or phenantroline based SiNN proligands (**L3**-H–**L6**-H, Figure 3) were synthetized and tested. These compound serve as suitable ligands for the iridium-catalyzed borylation of C(sp^3^)–H bonds using bis(pinalcolato)diboron (Bpin)_2_ as boron source (12 examples) The authors found that the best performance was achieved using **L6**-H (4.0 mol%) as the proligand, [Ir(Bpin)_3_(η^6^-mesitilyne)] (4.0 mol%) as the metallic precursor, 20 equiv of the corresponding alkane and 1.0 equiv of (Bpin)_2_, and heating at 120 °C for 20 h. Interestingly, addition of 6-isobutyl-2,2′-bipyridine, which is a carbon analog of SiNN pincer ligands, produced only a small amount of borylated product. These results clearly show that the silyl group is important for high efficiency.

**SCHEME 3:**
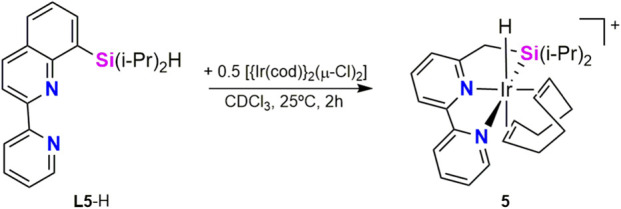
Synthesis of the Ir-κ^3^-*Si*,*N*,*N*′ complex **5**.

^1^H NMR studies on the reaction of **L5**-H with [{Ir(cod)}_2_(μ-Cl)_2_] revealed the quantitative formation of [Ir(H)(κ^3^-*Si*,*N*,*N′*-**L5**)(cod)]Cl (**5**), which was characterized by NMR spectroscopy and high-resolution mass-spectrometry (HRMS) (Scheme 3). The ^1^H NMR (CDCl_3_) spectrum of **5** exhibits a singlet resonance at δ −11.6 ppm, which is assigned to the Ir–H moiety. Borylation of C(sp^3^)–H bonds proceed using **5** as catalyst and even using [IrCl(cod)] (cod = cyclooctadiene) as iridium precursor and **L5-H**. On the basis of these observations, Kuninobu, Torigoe and Kawazu proposed that the catalytic active species corresponds to an iridium(III) complex of the type [Ir(Bpin)_2_(κ^3^-*Si*,*N*,*N′*-**Ln**)] (n = 3, 4, 5, or 6) (Kawazu et al., 2022).

This proposal was subsequently challenge by Su, Zhong, and coworkers, who reported a comprehensive density functional theory (DFT) study in 2024 on Ir-catalyzed C(sp^3^)–H borylation mediated by Ir-(κ^3^-*Si*,*N*,*N′*-**L6**) complexes (Bao et al., 2024). Their calculations indicate that the reaction mechanism can be divided into two key stages: (i) identification of a pentavalent iridium species, [Ir(H)(bpin)_3_(κ^3^-*Si*,*N*,*N′*-**L6**)], as a resting state and (ii) C(sp^3^)–H borylation mediated by the active intermediate [Ir(bpin)_2_(κ^3^-*Si*,*N*,*N′*-**L6**)]. The rate-determining step was identified as C–H oxidative addition to active catalytic species [Ir(bpin)_2_(κ^3^-*Si*,*N*,*N′*-**L6**)], while the high catalytic activity was attributed to the thermodynamic instability of the Ir(V) resting state intermediate, which readily converts into the active species, in contrast to the non-silyl containing species [Ir(bpin)_3_(κ^2^-*N*,*N′*)] formed from [Ir(bpin)_5_(κ^2^-*N*,*N′*)]. Notably, the SiNN-pincer ligand **L6** facilitates C(sp^3^)–H activation and hydride migration and stabilizes a key Ir(V) dihydride intermediate, enabling efficient borylation with boranes (Bao et al., 2024).

**SCHEME 4:**
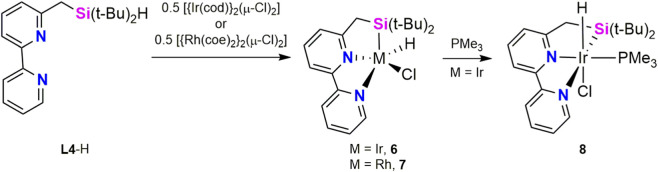
Synthesis of the Ir-**L4** complexes **6** and **8** and the Rh-**L4** species **7**.

At the same time than Kuninobu *et al.* published that LLX-type mer-tridentate ligands with silyl donor groups were suitable for borylation of C(sp^3^)–H bonds (Kawazu et al., 2022), Tobita *et al.* reported synthesis and catalytic activity in C–H borylation of arenes of Rh and Ir complexes bearing a SiNN ligand (Komuro et al., 2022b). Thus, reaction of **L4**-H with 0.5 equiv of the corresponding metal precursor, [{Ir(cod)}_2_(μ-Cl)_2_] or [{Rh(coe)_2_}_2_(μ-Cl)_2_], affords the hydride complexes [M(H)(Cl)(κ^2^-*Si*,*N*,*N′*-**L4**)] (M = Ir, **6**; Rh, **7**) complex. Treatment of **6** with PMe_3_ yields the Ir(III) derivative [Ir(H)(Cl)(*mer*-κ^2^-*Si*,*N*,*N′*-**L4**)(PMe_3_)] (**8**) (Scheme 4). The X-ray structures of **6** and **7** show the geometry around the metal center as highly distorted square pyramidal with the Si atom in the apical position for both complexes. This distortion was attributed to the competition between the geometrical restrictions of the pincer ligand and the strong trans effect of the silyl ligand giving a intermediate between *fac*- and *mer*- coordination modes of the pincer ligand. Dynamic behavior of the ligand in solution was also observed by variable-temperature NMR studies of **6** and **7** in THF-d_8_, which confirmed an equilibrium between the *fac*- and *mer*-coordination modes of **L4** ligand. The ^1^H NMR (C_6_D_6_, r.t.) spectra of complexes **6** and **7** display the metal-hydride resonance at δ −17.94 (s) and −16.00 ppm (br d, ^1^ *J*_Rh-H_ = 29.8 Hz), respectively. In the case of **7** and **8**, Si···H interaction is noticeable as the hydride resonance shows ^29^Si satellites (^1^ *J*_Si-H_ = 33 Hz) for **7** and the ^1^H NMR (CD_2_Cl_2_, r.t.) spectrum of **8** shows the Ir–H resonance as a doublet at −23.73 ppm (^2^ *J*_H-P_ = 22.8 Hz), accompanied by ^29^Si satellites (^1^ *J*_Si-H_ = 8.2 Hz) (Komuro et al., 2022b).

The catalytic activities of complexes **6**, **7** and **8** in the dehydrogenative borylation of benzene using B_2_Pin_2_ or HBpin were evaluated under neat conditions and at different temperatures, the best performance was achieved at mild conditions (40 °C) with a catalyst loading of 1.0 mol%. Among these catalysts, the iridium derivative **6** exhibited superior catalytic performance relative to complexes **7** and **8**. To assess the scope of the reaction, the dehydrogenative borylation catalyzed by **6** (1.0 mol%) was extended to substituted arenes, including toluene, bromobenzene and fluorobenzene. These substrates were successfully borylated to afford the corresponding *ortho*:*metha*:*para* isomers in 93%, 99% and 94% yield based on boron, respectively (Komuro et al., 2022b). The proposed mechanistic cycle also involves a bis(boryl)silyl complex [Ir(bpin)_2_(κ^3^-*Si*,*N*,*N′*-**L4**)] as the catalytic active species as the alternative of the proposed tris(boryl) iridium intermediate assumed for the Ir-bipy system (Figure 2A). The design of the ligand is crucial for the success of the reaction as the strong σ-donor character of the silyl ligand promotes the activation of the C–H bond of the arenes and the flexibility of the system is involved in lowering the activation energy of this step likely by decreasing the steric hindrance in the transition state of this oxidative addition.

### 2.2 Deuteration catalysts

**SCHEME 5:**
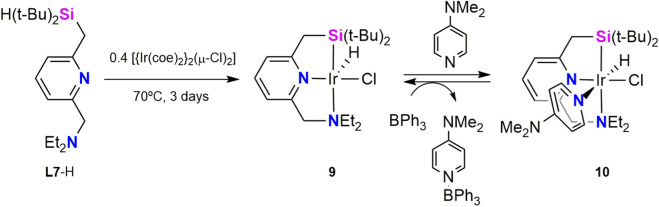
Synthesis of the Ir-**L7** complexes **9** and **10**.

In 2019, Tobita et al. reported the synthesis of iridium complexes bearing a tridentate silyl–pyridine–amine pincer ligand. They hypothesized that an amine moiety *trans* to a strong σ-donor silyl ligand could facilitate the C–D bond activations because a) strong *trans* influence would promote hemilability of the amine ligand to generate a coordinatively unsaturated catalytic species, and b) strong σ-donating properties of the silyl ligand would generate an electron-rich metal center and facilitate oxidative addition to it. These complexes were prepared from *N*-((6-((dimethylsilyl)methyl)pyridin-2-yl)methyl)-N-ethylethanamine (**L7**-H) as proligand and were shown to be active catalysts for the deuteration of hydrosilanes using C_6_D_6_ as the deuterium source (Komuro et al., 2019). Complex [Ir(H)(Cl)(κ^3^-*Si*,*N*,*N′* **-L7)**] (**9**) was obtained by reaction of **L7**-H with 0.4 equiv of [{Ir(coe)_2_}_2_(μ-Cl)_2_] at 70 °C in toluene for 3 days in 75% yield. Treatment of **9** with one equiv of 4-dimethyaminopyridine (DMAP) at r.t. led instantaneously to the formation of complex [Ir(H)(Cl)(κ^3^-*Si*,*N*,*N′* **-L7**)(DMAP)] (**10**). Complexes **9** and **10** are interconvertible, thus the reverse conversion of **10** to **9** was achieved by reaction with one equiv of the Lewis acid BPh_3_ at r.t. (Scheme 5). Complexes **9** and **10** were fully characterized by NMR spectroscopy and X-ray diffraction analysis. The solid-state structure of **9** reveals two crystallographically independent molecules with Ir–Si bond distances of 2.2991(12) and 2.3001(13) Å. These values are comparable to the Ir–Si bond distance of 2.2938(11) Å observed for **10** and fall within the typical range reported for Ir–silyl bond (Batuecas et al., 2024; Padilla et al., 2025a). Ir–H bond in complex **9** is tilted towards the Si atom with an average Si-Ir–H angle of 60°, suggesting a Si···H interaction in solid state, also supported by DFT optimization of the geometry. On the other hand, complex **10** exhibits dynamic behavior in solution, caused by the dissociation of the amine ligand supporting the hemilability of the **L7** pincer ligand.

Complexes **9** and **10** were shown to be active catalysts for the deuteration of tertiary hydrosilanes at room temperature, with the 16-e unsaturated species **9** being significantly more active than **10** (Komuro et al., 2019). The reaction rate of the deuteration depends on both electronic and steric factors. In the deuteration of hydrosilanes HSiMe_2_R (R = Et, *i*-Pr, *t*-Bu, Ph), reation rates decrease with the bulkiness of the alkyl substituent in the order Et > *i*-Pr > *t*-Bu, being the reaction with R = Ph markedly slower. Notably, highly sterically hindered Si–H bonds, such as those in HSiEt_3_ and HSi(*n*-Pr)_3_ performed well in the deuteration reaction while siloxy-substituted tertiary silanes HSiMe(OSiMe_3_)_2_, and the dihydrosilane (H_2_SiEt_2_) gave low or undetectable deuterated product.

**SCHEME 6:**
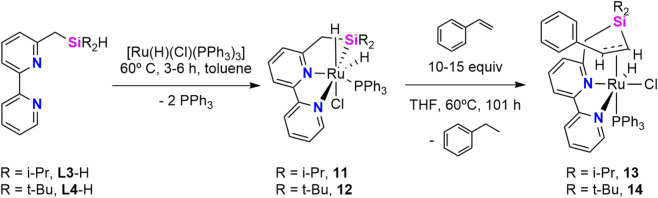
Synthesis of the Ru-(κ^3^-*Si*,*N*,*N′*) complexes **11**, **12**, **13** and **14**.

Same group investigated the applicability of κ^3^-*Si*,*N*,*N* pincer ligands for catalytic C(alkenyl)–H deuteration with C_6_D_6_. Seven coordinated ruthenium complexes bearing silyl ligands **L3** and **L4** were synthetized and have been found to be active catalysts for the deuteration of olefins (Komuro et al., 2025). Reaction of the corresponding proligand, **L3**-H or **L4**-H, with [Ru(H)(Cl)(PPh_3_)_3_] leads to the formation of the corresponding dihydride Ru(IV) [Ru(H)_2_(Cl)(κ^3^-*Si*,*N*,*N′*-**Ln**)(PPh_3_)] (**Ln** = **L3**, **11**; **L4**, **12**) (Scheme 6). Both complexes were characterized by NMR and X-ray spectroscopy. Crystal structures in both molecules adopt a seven coordinated distorted octahedral geometry, with Ru–Si bond distances 2.3619(11) Å (**11**) and 2.3924(7) Å (**12**), in the range of typical Ru–silyl bond lengths (Corey, 2016). The two Si···H interatomic distances imply a weak secondary interaction, which was supported by DFT calculations on an optimized model molecule. The ^1^H NMR (CD_2_Cl_2_, r.t.) spectra of **11** and **12** exhibit signals assigned to the two different hydride ligands centered at δ −14.78 and −12.19 ppm (**11**) and at δ −14.62 and −12.09 ppm (**12**). In the case of complex **12**, the signal at δ −14.62 ppm displays satellites arising from ^29^Si coupling (^1^ *J*_Si-H_ = 22.5 Hz), indicating that a weak Si···H interaction exist also in solution.

Treatment of complexes **11** and **12** with excess (15 equiv) of styrene in THF at 60 °C formed complexes **13** and **14**, respectively, together with ethylbenzene (Scheme 6). The proposed structures were confirmed by single crystal X-ray diffraction analysis of **14**. The ^1^H NMR (CD_2_Cl_2_, r.t.) spectra of **13** and **14** exhibit signals assigned to the hydride ligands centered at δ −7.96 ppm (**13**) and at δ −7.13 ppm (**14**). The ^29^Si{^1^H} NMR (CD2Cl2, IG, r.t.) spectra displays the resonance at δ 7.2 (**13**) and 6.8 ppm (**14**), these values are highly upfield-shifted compared to the values observed for **11** and **12** and consistent with the absence of Ru-Si bonds.

With the Ru-(κ^3^-*Si*,*N*,*N′*) complexes **11** and **12** in hands, the authors investigated the catalytic deuteration of monosubstituted olefins with C_6_D_6_. In the presence of 10 mol% of **11**, bearing less sterically demanding i-Pr substituents at silicon, efficient and highly selective alkenyl C–H deuteration was achieved at 60 °C for a range of olefins, including styrene derivatives and tert-butylethylene. In contrast, the bulkier analogue **12**, containing t-Bu groups on silicon, showed minimal catalytic activity, indicating that steric congestion at the silicon center severely hampers C–H activation. The reactions catalyzed by **11** afforded triply deuterated olefins (R′CD═CD_2_) with high deuterium incorporation (92%–99%), while no competing deuteration at aryl or alkyl C–H sites was observed, underscoring the excellent chemoselectivity of the system.

During styrene deuteration catalyzed by **11**, the formation of complex **13** was detected by ^1^H NMR spectroscopy. Complex **13** enabled deuteration, although at a significantly reduced rate compared to **11** suggesting that **13** is not an active species but rather a resting state in the catalytic cycle. Furthermore, the addition of PPh_3_ to reactions catalyzed by **11** markedly suppressed both reaction rate and deuterium incorporation, indicating that phosphine dissociation from ruthenium constitutes the turnover-limiting step. According to this experimental observations, a catalytic cyclic in which the C–D oxidative addition of C_6_D_6_ to an alkene coordinated [Ru-(κ^3^-*Si*,*N*,*N′-* **L3**)(Cl)(η^2^-alkene)PPh_3_] intermediate along with phosphine dissociation was proposed as the rate limiting step. The nature of the SiNN ligand **L3** facilitates key steps of the proposed catalytic cycle. The skeletal flexibility of the ligand allows formation of congested seven-coordinated intermediates and migratory insertion of the styrene into Ru–D bond. Furthermore, the strong σ-donor character of the silyl ligand electronically enriches the ruthenium center, thereby lowering the energy barrier for the rate-limiting C–D oxidative addition of the C_6_D_6_ to the electron-rich metal center.

## 3 Transition-metal complexes with κ^3^-(*Si*,*N*,*P)* ligands

### 3.1 C–H borylation catalysts

**FIGURE 4:**
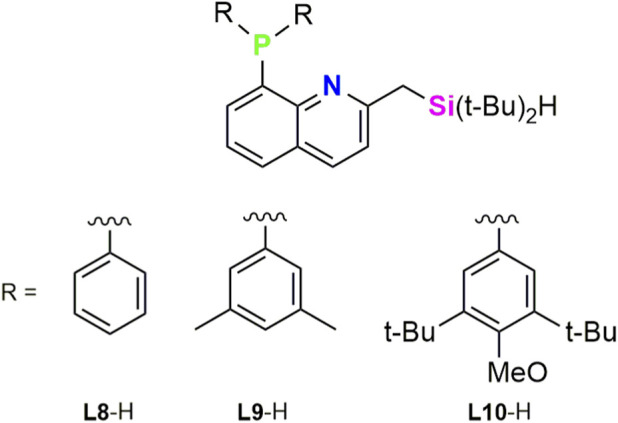
SiNP ligands reported by Torigoe, Ohmura, and collaborators.

Based on the results reported of the Ir/SiNN catalyzed borylation of C(sp^2^)–H (SiNN = **L4**, Komuro et al., 2022b) and C(sp^3^)–H (SiNN = **L3** – **L6**, Kawazu et al., 2022), in 2025, Torigoe, Ohmura, and co-workers reported quinoline-based XLL-type mer-tridentate SiNP ligands and applied them in the Ir-catalysed C(sp^3^)–H borylations (Torigoe et al., 2025). With the aim of increasing the scope of the chemoselectivity conversion of C(sp^3^)–H in the presence of C(sp^2^)–H, a series of *mer*-tridentate SiNP ligands with bulky substituents at the coordination sites (**L8** – **L10**) were designed by Torigoe and Ohmura group. These ligands combine a hydrosilylmethyl group and a diarylphosphino moiety, enabling efficient *mer*-coordination and enhanced catalyst chemoselectivity by increasing sensitivity at hindrance in the reactive site (Figure 4).

Compounds **L8**-H, **L9**-H and **L10**-H were used as proligands for the iridium catalyzed C(sp^3^)–H borylation using [{Ir(cod)}_2_(µ-OMe)_2_] as metallic precursor and bis(pinacolato)diboron as boron source. These studies revealed that electron-rich and bulky phosphine substituents are critical, with ligand **L10**-H delivering exceptional activity. The catalytic system based on Ir/**L10** exhibits broad substrate scope, displaying pronounced site- and chemoselectivity, favoring less hindered primary bonds over secondary positions and even over competing C(sp^2^)–H bonds. This selectivity was demonstrated and applied in the late-stage functionalization of a liquid crystal material. Overall, these SiNP ligands provide a powerful platform for controlling reactivity and selectivity in iridium-catalyzed C(sp^3^)–H borylation without directing groups, with steric congestion around the metal center emerging as a key determinant of C(sp^3^)–H versus C(sp^2^)–H selectivity. The authors propose that the notable chemoselectivity of the Ir/SiNP systems, in comparison with the previously reported Ir/SiNN catalysts, may be due to the sterically congested environment around the iridium center created by the bulky substituents on the phosphorus atom of the SiNP ligands (Torigoe et al., 2025).

## 4 Transition-metal complexes with κ^3^-(*Si*,*Si’*,*P)* ligands

### 4.1 C–H silylation catalysts

**SCHEME 7:**
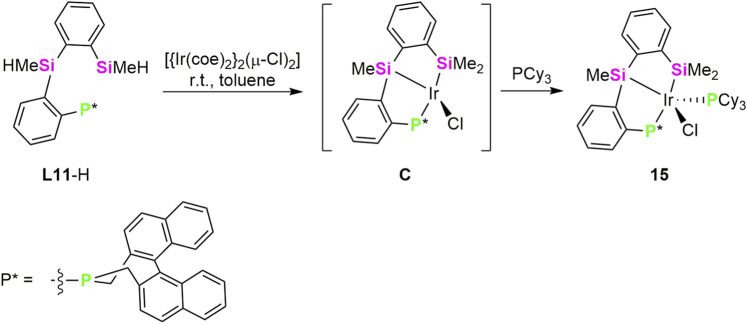
Synthesis of the Ir-(κ^3^-*Si*,*Si’*,*P*-**L11**) complex **15**.

In 2023, Ge, He and collaborators reported the Ir-catalysed atroposelective intermolecular C–H silylation enabled by a chiral (κ^3^-*Si*,*Si’*,*P*) ligand. The design of the ligand is based on the previously mentioned geometry of pincer ligands with chelate effect which inhibit decomposition of catalytic species and the strong σ-donor character of silyl fragment which favors the oxidative addition of the C–H bond to the electron-rich metal centre. Thus, authors synthetised a (κ^3^-*Si*,*Si’*,*P*) proligand (**L11-**H) (Scheme 7), in which the incorporation of a silyl ligand into an axially chiral scaffold provide excellent chemo-, regio-, and stereoselective in the C–H silylation of a variety of 1-arylisoquinolines (Yang et al., 2023).

**SCHEME 8:**
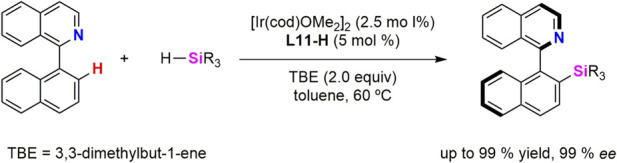
Ir/**L11-H**-catalyzed atroposelective C-H silylation.

The catalytic system [Ir(cod)}_2_(µ-OMe)_2_]/**L11**-H has proven to be effective for atroposelective intermolecular C–H silylation of 1-arylisoquinolines in toluene at 60 °C (Scheme 8). The reaction displays a broad substrate scope, tolerating diverse functional groups and π-extended frameworks, and delivers axially chiral silanes in generally high yields and excellent enantioselectivities. Both aryl- and alkyl-substituted silanes are compatible, whereas highly substituted hydrosilanes are ineffective under the standard conditions. The synthetic utility of the method was demonstrated through stereospecific derivatizations, enabling access to a range of axially chiral building blocks and chiral NO-chelating ligands. Overall, this work introduces a new class of chiral silyl pincer ligands and establishes an efficient strategy for Ir–catalyzed atroposelective intermolecular C–H silylation, thereby expanding the toolbox of asymmetric organosilicon chemistry (Yang et al., 2023).

Willing to know about the reaction mechanism, reaction of the proligand **L11**-H with 0.5 equiv of the iridium precursor, [{Ir(coe)_2_}_2_(µ-Cl)_2_] or [{Ir(cod)}_2_(µ-OMe)_2_] was carried out without isolation of any complex. However, when same reaction was tried in the presence of one equiv of PCy_3_, the iridium(III) species [Ir(Cl)(κ^3^-*Si*,*Si’*,*P*-**L11**)(PCy_3_)] (**15**) was obtained in 76% yield (Scheme 7). The authors proposed **C** as the unisolable intermediate in the formation of **15**. Complex **15** was characterized by ^1^H, ^13^C and ^31^P NMR spectroscopy and by single-crystal X-ray diffraction. The Ir–Si bond distances in **15** (2.331.(2) and 2.330(2) Å) fall within the range of Ir–silyl bond distances (Batuecas et al., 2024; Padilla et al., 2025a). Mechanistic studies support the involvement of an unsaturated Ir–silyl species (**C** in Scheme 7) as the active catalyst, with C–H bond activation identified as the rate-determining step (Yang et al., 2023).

**SCHEME 9:**
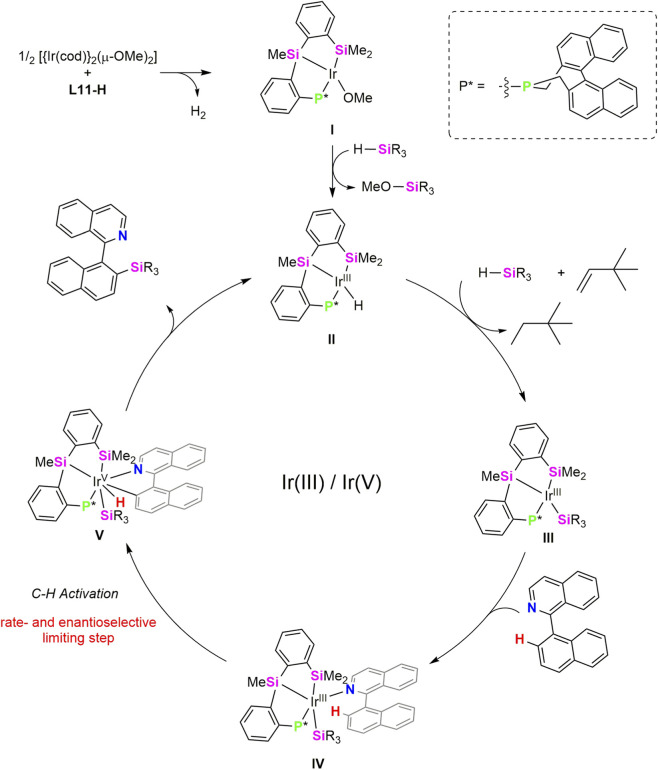
Catalytic proposed cycle for Ir/**L11-H**-catalyzed atroposelective C-H silylation.

A mechanistic Ir(III)-Ir(V) pathway was proposed and deeply investigated by DFT calculations few years later by the same group (Pan et al., 2026). The catalytic species is formed by reaction of **L11**-H with [{Ir(cod)}_2_(µ-OMe)_2_], to give intermediate (**I**), related to **C** in Scheme 7. A σ-bond methatesis with the hydrosilane produces a hydride intermediate (**II**), proposed to be the active catalytic species. Then insertion of TBE (TBE = 3,3-dimethylbut-1-ene), used as the hydrogen acceptor, into the Ir–H bond followed by Si–H oxidative addition and reductive elimination of the hydrogenated-TBE (2,2-dimethylbutane) gives Ir(III) –silyl intermediate (**III**), this transformation was found to be exergonic by 24.9 kcal mol^−1^. Finally, C(sp^2^)–H oxidative addition into the substrate coordinated intermediate **IV** to form a Ir(V)-silyl intermediate (**V**) and C–Si reductive elimination release the silylated product (Scheme 7) regenerating the hydride intermediate **I**. The C(sp^2^)–H oxidative addition step was found to be both rate- and enantioselectivity-determining step. Distortion/interaction and structural analyses reveal a ligand-enabled axial chirality transfer mechanism, in which the backbone of the chiral κ^3^-*Si*,*Si’*,*P*-**L11** pincer ligand generates an axially chiral environment that governs stereocontrol through a match/mismatch effect with the 2-arylisoquinoline substrate (Scheme 9).

## 5 Transition-metal complexes with κ^4^-(*Si*,*N*,*C–H)* ligands

Our group has recently published the synthesis of the XLL-type proligand (4,8-dimethylquinoline-2-yloxy)dimethylsilane (**L12**–H) which serves as an effective entry to the chemistry of agostic Ir-(κ^4^-*Si*,*N*,*C–H*) complexes, active catalysts for the solventless synthesis of silazanes via cross-dehydrogenative coupling (CDC) between secondary amines and hydrosilanes (Padilla et al., 2025b). The design of this ligand was inspired by the observation that the iridium Ir-(κ^2^-*Si*,*N*) complex [Ir(H)(OTf)(κ^2^-NSi^tBu^_2_)(PCy_3_)] (NSi^tBu2^ = 4-methylpyridin-2-yloxy-ditertbutylsilyl), recently reported (Gómez-España et al., 2022), exhibits moderate catalytic activity in the cross-dehydrogenative coupling (CDC) of *N*-methylaniline with hydrosilanes. To enhance this catalytic activity, new κ^2^-NSi ligands with reduced steric hindrance were designed by replacing tert-butyl groups with methyl substituents at the silicon atom. However, the use of these ligands led to the unavoidable coordination of two ligand units to the metal center. Willing to obtain an unsaturated metal complexes containing only one κ^2^-NSi ligand, we explored 2-quinolone derivatives as alternative proligands for iridium complexes. As a result, unsaturated species stabilized by Ir···H–C agostic interactions were successfully synthesized and were found to be active catalysts for the CDC of secondary amines with hydrosilanes.

**SCHEME 10:**
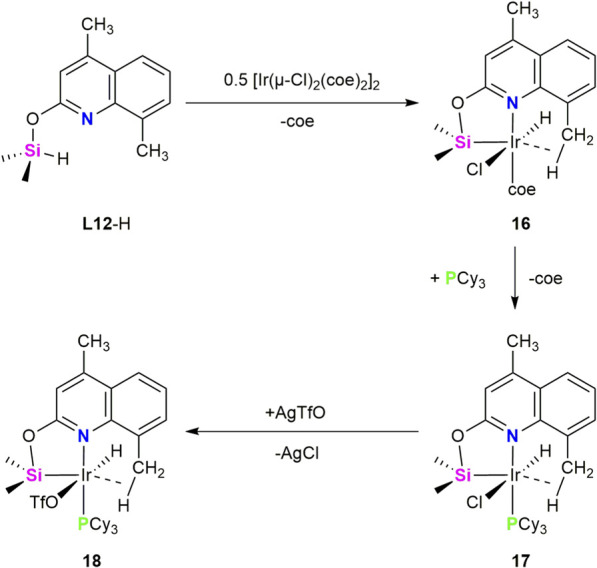
Synthesis of the Ir-(κ^4^-*N*,*Si*,*C*-*H*-**L12**) complex **16**, **17** and **18**.

Treatment of **L12**–H with 0.5 equiv of [{Ir(coe)_2_}_2_(μ-Cl)_2_] in CH_2_Cl_2_ at r.t. afforded the Ir(III) complex [Ir(H)(Cl)(κ^4^-*N*,*Si*,*C*-*H*-**L12**)(coe)] (**16**). Subsequent reaction of **16** with one equiv of PCy_3_ led to the corresponding phosphine derivative [Ir(H)(Cl)(κ^4^-*N*,*Si*,*C*-*H*-**L12**)(PCy_3_)] (**17**). Complex **17** reacted with one equivalent of AgOTf to give [Ir(H)(OTf)(κ^4^-*N*,*Si*,*C*-*H*-**L12**)(PCy_3_)] (**18**) (Scheme 10). Complexes **16**–**18** were characterized by elemental analysis and NMR spectroscopy, and complexes **17** and **20** were further confirmed by single-crystal X-ray diffraction. The ^29^Si NMR resonances at δ 32.8 (**16**), 28.2 (**17**) and 29.0 ppm (**18**) are downfield field relative to that of **L12**-H (δ 3.3 ppm). In conjunction with the signals observed in the ^1^H NMR spectra at −16.64 (**16**), −20.87 (d, ^2^ *J*_HP_ = 18.8 Hz) (**17**) and −29.18 ppm (d, ^2^ *J*_HP_ = 19.2 Hz) (**18**), these data support the oxidative addition of the Si–H bond to the iridium center. The presence of an agostic Ir···H–C interaction between the iridium center and one of the C–H bonds of the 8-CH_3_ substituent on the quinolone ring was confirmed by NMR spectroscopy, X-ray diffraction analysis and DFT calculations (Padilla et al., 2025b). The Ir–Si bond distances in complexes **17** (2.2529(4) Å) and **18** (2.2687(4) Å) are shorter than those reported for the related 2-pyridone derivatives [Ir(H)(X)(κ^2^-NSi^tBu2^)(PCy_3_)] (X = Cl, OTf), which are approximately 2.28Å (Gómez-España et al., 2022). In this regards, we have recently reported a comprehensive analysis of Ir–Si bonding in a range of iridium–silyl and iridium–silylene complexes using NBO and QTAIM methodologies. The results of this studies show that to define the nature of the Ir–Si bond in complexes **16**, **17** and **18**, it is necessary to study the environment of the Si atom. In these complexes, the Si–O interaction shows a dative O→Si bonding mode, as revealed through low WBI values and strong donor-acceptor interactions from oxygen to silicon, supported by second-order perturbation theory. Notably, the same computational protocol was applied by us to other complexes that are unambiguously described in the literature as base-stabilized silylenes. In those cases, the electronic descriptors (WBI values, donor–acceptor interaction energies, and charge distribution) clearly supported a silylene formulation. The close similarity between those benchmark systems and complexes **16**–**18** strongly supports the assignment of a base-stabilized silylene character to the Ir–Si bond in these complexes (Padilla, et al., 2025a).

Complexes **16**–**18** have shown to be active catalysts for the solventless synthesis of silazanes *via* cross-dehydrogenative coupling (CDC) between secondary amines and hydrosilanes. Among them, complex **18** exhibits significantly higher catalytic activity than complexes **16** and **17**. Notably, complex **18** acts a versatile and highly efficient catalyst precursor for the CDC of secondary amines with HSiMe_2_Ph. Remarkably, it also ranks among the most active catalysts reported for the CDC of the more challenging *N*-alkylaniline derivatives with HSiMe_2_Ph, displaying TOF_1/2_ values in the range of 8.4 10^3^ to 7.9 10^4^ h^-1^ (Padilla et al., 2025b).

## 6 Conclusion and future challenges

This review highlights the central role of asymmetric tridentate organosilyl ligands as enabling platforms in homogeneous transition-metal catalysis, with most reported examples involving iridium and rhodium complexes and a more limited number of cases based on ruthenium, emphasizing how the presence of a peripheral silicon center influences both structure and reactivity. These Si-containing frameworks display non-innocent behavior, engaging directly in metal–ligand cooperation through reversible Si–H activation, σ-complex formation, agostic interactions, and hydride stabilization. The silicon substituents modulate the electronic properties of the metal center, stabilize high-valent and unsaturated intermediates, and lower activation barriers for challenging bond-activation processes, thereby enhancing catalytic efficiency and selectivity.

A recurring theme across κ^3^-(Si,N,N′), κ^3^-(Si,N,P), κ^3^-(Si,Si’,P), and κ^4^-(Si,N,C–H) coordination modes is the ability of the peripheral silicon atom to act as a dynamic relay for hydride and substituent migration, effectively coupling ligand flexibility with catalytic turnover. In borylation, deuteration, and silylation reactions, this cooperativity enables access to multiple low-energy pathways, mitigates deactivation, and governs chemo-, regio-, and stereoselectivity. Notably, subtle variations in ligand rigidity, steric demand at silicon, and coordination geometry (*fac* vs. *mer*) translate into dramatic changes in catalytic performance, underscoring the importance of precise ligand design in exploiting silicon-enabled reactivity.

These catalysts have been most frequently applied in C(sp^3^)–H borylation, with additional examples enabling selective deuteration, and dehydrogenative silylation, demonstrating their growing impact across diverse catalytic manifolds. Looking forward, continued integration of experimental and computational approaches will be crucial to fully harness the potential of peripheral silicon effects, enabling the rational development of next-generation catalysts for sustainable, selective, and mechanistically sophisticated transformations.

Despite these advances, the chemistry of transition-metal complexes supported by asymmetric tridentate organosilyl ligands remains at an early stage, and numerous opportunities exist for further development. Particularly promising directions include the systematic modulation of the silicon environment thorough substituent effects and donor–acceptor interactions with heteroatoms. Fine-tuning these parameters is expected to provide precise control over metal–silicon bonding, ligand hemilability, and hydride stabilization, thereby enabling catalyst optimization for specific bond-activation processes.

From a mechanistic standpoint, deeper insight into silicon-enabled metal–ligand cooperativity will be essential. While density functional theory has already elucidated the roles of Si–H activation, Bpin migration, and hydride shuttling, future studies combining operando spectroscopy, isotopic labeling, and advanced computational methods could clarify how peripheral silicon governs selectivity-determining steps and catalyst resting states.

Finally, the integration of asymmetric organosilyl ligands into new catalytic manifolds and sustainable transformations represents an exciting Frontier. Applications in challenging small-molecule activation, late-stage functionalization, electrocatalysis, and isotope-labeling technologies appear especially promising. Moreover, extending these ligand platforms to earth-abundant metals could significantly broaden their impact and address sustainability concerns. Overall, continued exploration of peripheral silicon effects is poised to reshape ligand design strategies, positioning asymmetric organosilyl pincers as key components in the next-generation of cooperative and selective transition-metal catalysts.
